# Full-Length Transcriptome Sequencing and the Discovery of New Transcripts in the Unfertilized Eggs of Zebrafish (*Danio rerio*)

**DOI:** 10.1534/g3.119.200997

**Published:** 2019-03-23

**Authors:** Rumana Mehjabin, Lv Xiong, Rong Huang, Cheng Yang, Geng Chen, Libo He, Lanjie Liao, Zuoyan Zhu, Yaping Wang

**Affiliations:** * State Key Laboratory of Freshwater Ecology and Biotechnology, Institute of Hydrobiology, Chinese Academy of Sciences, Wuhan 430072, China; † University of Chinese Academy of Sciences, Beijing 100049, China

**Keywords:** full-length transcriptome sequencing, unfertilized egg, RNA-seq, zebrafish (*Danio rerio*)

## Abstract

Understanding early gene expression in zebrafish embryos is a prerequisite for developmental biology research. In this study, 1,629,447 polymerase reads were obtained from the unfertilized eggs of zebrafish via full-length transcriptome sequencing using the PacBio RS II platform first. Then, 102,920 unique isoforms were obtained by correction, clustering and comparison with the zebrafish genome. 12,782 genes in the genome were captured, accounting for 39.71% of the all annotated genes. Approximately 62.27% of the 12,782 genes have been alternatively spliced. GO and KEGG annotations revealed that the unfertilized eggs primarily stored genes that participate in RNA processing and nuclear protein complex composition. According to this PacBio data that aligned with the genome, 3,970 fusion genes, 819 ncRNAs, and 84 new transcripts were predicted. Illumina RNA-seq and RT-qPCR detection found that the expression of two new transcripts, PB.5289.1 and PB.10209.1, were significantly up-regulated at the 2-cell stage and down-regulated rapidly thereafter, suggesting their involvement in minor ZGA during early embryonic development. This study indicated that the unfertilized eggs of zebrafish may have retained genes directly related to cell division and development to initiate the subsequent development in a limited space and time. On the other hand, NTRs or new transcriptome regions in the genome were discovered, which provided new clues regarding ZGA of MZT during early embryonic development in fish.

In recent years, the development of high-throughput sequencing technology has made transcriptome sequencing possible, becoming the primary method to study the regulation of gene expression (Gusev *et al.* 2016). Transcriptome research has flourished using third generation sequencing platforms such as the PacBio, which can directly read full-length transcript without interruption. It can effectively obtain high-quality sequence of a single RNA molecule in its entirety and accurately identify the transcripts of differentially expressed isoforms, homologous genes, superfamily genes, and alleles that are not recognized by next generation sequencing (NGS). It can improve the real-time quantitative polymerase chain reaction (RT-qPCR) results of gene expression, identify the phenomena of alternative splicing (AS) and gene fusion, and discover new genes and transcriptional isomers (Wang *et al.* 2016; Abdel-Ghany *et al.* 2016).

Zebrafish are often used as model animals for developmental biology studies including the mechanisms of maternal mRNA expression and gene network regulation because of their short cell cycle, synchronous embryonic development, high spawning capacity, etc. (Yu *et al.* 2001). Embryonic development begins with a single fertilized egg cell formed by the ovum and sperm. All cytoplasm comes from the female ovum and the nucleus results from the fusion of the male and female primitive nucleus. At the initial stage of development, the genomic transcription of the embryo is static and embryonic development is directed entirely by maternal RNA and proteins (Tadros and Lipshitz 2009). As embryonic development proceeds, the maternal factor in the control of the developmental process is gradually transferred to the zygote, which then assumes control of gene production. This process is called maternal-to-zygotic transition (MZT). At this point, maternal factors are gradually cleared and zygotic genome activation (ZGA) and transcription begin (Tadros and Lipshitz 2009). Full-length transcriptome sequencing of unfertilized eggs of zebrafish is of great significance in revealing the expression pattern of maternal mRNA and the regulatory mechanisms of early embryonic development.

It is well known that the expression of genes during development must conform to precise spatial and temporal regulation; therefore, the developmental process is more complex and rigorous than other life processes (Lee *et al.* 2014). Alternative splicing (AS), as a way of post transcriptional processing, is an important step in the process of gene expression regulation. In different tissues, cells, or developmental stages in eukaryotes, the AS types of many genes are not exactly the same. Once transcribed into mature mRNA, genomic DNA has not undergone a one-time removal all of introns, but rather a selective excision of introns at different splice sites; at the same time, the roles of isomers of the same gene may also be different (Mittendorf *et al.* 2012; Lu *et al.* 2012). Although many studies have investigated the changes in expression levels of various genes during the development of zebrafish, most of them are based on the NGS methods (Aanes *et al.* 2011; Vesterlund *et al.* 2011; Aanes *et al.* 2013). Furthermore, an abundance of work remains in seeking out new AS and transcripts.

The entire life cycle of an organism involves dynamic development. The study of the means by which a single-cell fertilized egg, through a series of cell divisions and differentiation, produces an organism with cells of different shapes and functions, as well as the ability of these cells to construct various tissues and organs through the interaction between cells that build an organism by completing various developmental processes, must be expanded. Zebrafish is not only an ideal model for the study of developmental biology (Dooley and Zon 2000), it is also an ideal model for the study of the genetic basis of human diseases (Chandrasekar *et al.* 2013). However, the annotation of the zebrafish genome is incomplete and remains a work-in-progress. Therefore, the PacBio RS II sequencing data of zebrafish provide an opportunity for the excavation of novel transcribed regions (NTRs).

Several transcriptome analyses have been performed in zebrafish by using RNA-Seq (Aanes *et al.* 2011; Vesterlund *et al.* 2011; Pauli *et al.* 2012; Harvey *et al.* 2013; Nepal *et al.* 2013). In addition to the dynamic analysis of transcriptome data from the early developmental stages of embryos, NTRs in annotated and non-annotated regions of some zebrafish genomes have also been described (Aanes *et al.* 2011; Vesterlund *et al.* 2011; Aanes *et al.* 2013). The main purpose of this study is to understand the gene expression patterns in the early embryonic development of zebrafish using PacBio RS II sequencing first and to discover new transcripts in the zebrafish genome. The results here provide new candidates that when functionally investigated may provide new insights into the ZGA of MZT during early embryonic development in zebrafish.

## Materials and Methods

### Collection of unfertilized eggs and PacBio RS II sequencing of zebrafish

Three female zebrafish were selected and 100 unfertilized eggs from each female were harvested as three samples (Z1-1, Z1-2, and Z1-3) respectively. The remaining eggs were incubated at 28° after fertilization and 100 fry were taken as three samples (Z2-1, Z2-2, and Z2-3) 24hr. after fertilization (∼ prim-5 stage). Trizol reagent (Invitrogen, Carlsbad, CA, USA) was used to extract the total RNA from each sample; 1% agarose gel electrophoresis and an ultramicro spectrophotometer (NanoDrop 2000, Thermo Fisher Scientific, Waltham, MA, USA) were used to detect the quality and concentration of each RNA sample.

2μg RNA from Z1-1, Z1-2, and Z1-3 were combined into sample Z1-M (NCBI BioSample No. SAMN10290469) which was reverse transcribed into cDNA using a SMARTer PCR cDNA Synthesis Kit (Clontech, Mountain View, California, USA). Four fragment ranges (0.5-1K, 1-2K, 2-3K, and >3K) were prepared according to the PacBio Iso-Seq protocol (Eid *et al.* 2009). PacBio RS II sequencing reactions of 16 SMRT (single-molecule real-time) cells (3 cells of 0.5-1K, 5 cells of 1-2K, 5 cells of 2-3K, 3 cells of >3K) were performed in DNA Sequencing Reagent 4.0 (Clontech, Mountain View, California, USA). The raw sequencing data (NCBI BioProject No. PRJNA498365) was filtered to obtain clean data. We used the multiple sub-reads obtained from the same polymerase sequencing reads for the self-correction of the clean data, thereby obtaining high-quality insert reads.

### Transcripts classification, clustering, and correction

Full-length reads were obtained by classifying the reads of inserts >300bp by judging the presence and location relationships of the 5′ primer, 3′ primer, and poly A regions. Transcriptome sequencing generated significant isoform redundancy. The iterative clustering and error correction (ICE) (Eid *et al.* 2009) algorithm module in the ICE toolkit was used to cluster the above redundant full-length reads together to yield the new full-length reads. The insertion sequence of the incompletely sequenced reads was then aligned back to the new full-length reads using Quiver to correct the mistakes and further enhance the accuracy of the isoform sequences. Isoforms with degrees of accuracy ≥99% were considered high quality isoforms (HQ isoforms) and those with lower degrees of accuracy (<99%) were considered low quality isoform (LQ isoforms).

### Comparison of HQ and LQ isoforms With reference genomes and gene prediction

HQ and LQ isoforms were compared with zebrafish reference genomes (ftp://ftp.ensembl.org/pub/release-86/fasta/danio_rerio/dna/Danio_rerio.GRCz10.dna_sm.toplevel.fa.gz) using GMAP software (Wu and Watanabe 2005) for parameter selection uniqueness. The fusion_finder.py (in GMAP software) was used to extract two or more gene sequences from the alignment result and the sequence with a certain reads-support degree (with transcript coverage ≥10% for each gene, alignment coverage ≥99%, and the sequence length ≥10K of the intergene) were considered to be candidate fusion genes.

After deleting fusion genes, there were still a large number of redundant sequences in the cluster results; we went on to use the collapse_isoforms_by_sam.py (in GMAP software) to filter and remove redundancy for the matching results with min-identity of 0.9 and min-coverage of 0.85. Comparing the matching results with the original genome annotation information and the PacBio annotation results were merged with the original genome annotation results. We selected the best matching results from each isoform sequence for statistical analysis; the matching and non-matching genes in the reference genome were subjected to GO (http://www.geneontology.org) and KEGG (https://www.genome.jp/kegg/) enrichment analyses, respectively.

The ncRNA_pipeline process (https://bitbucket.org/arrigonialberto/lncrnas-pipeline) was used to predict lncRNA (long non-coding RNA) sequences for the isoforms that were matched to the genome but without annotation results. The remaining unpredicted sequences were matched to the zebrafish gene bank of NCBI (https://www.ncbi.nlm.nih.gov/genbank/) and Ensembl database (http://asia.ensembl.org/index.html), and un-annotated sequences were screened as new transcripts. GO, KEGG, COG (http://www.ncbi.nlm.nih.gov/COG), SwissProt (https://www.ebi.ac.uk/uniprot), TrEMBL (https://www.ebi.ac.uk/uniprot), Pfam (http://pfam.xfam.org/) and NR annotation (https://www.ebi.ac.uk/patentdata/nr) were used to predict the homology, conservation, domain and function of these new transcripts. CPAT (http://lilab.research.bcm.edu/cpat/) and TransDecoder (https://github.com/TransDecoder/TransDecoder/wiki) were used to predict the protein-coding potentials of these new transcripts.

### AS and gene structure optimization

HQ and LQ isoforms were matched with zebrafish reference genomes to obtain gff3 files which were matched to the cDNA region; those isoforms that were extracted from the gff3 files and were matched to the same gene were identified as an AS site. The number of alternative donor (AltD) sites, alternative acceptor (AltA) sites, alternative position (AltP) sites, exon skipping (ExonS) sites, and intron retention (IntronR) sites (Mittendorf *et al.* 2012) were counted, along with the AS events of known genes. The 5′ or 3′ end of the gene was extended by comparing the sequencing results and the existing gene annotation results, such that the new annotated gene exon fragment would outperform the reference gene region.

### RNA-seq and expression-calculation of new transcripts in unfertilized egg and prim-5 stage

The total RNA of the six samples described above (Z1-1, Z1-2, Z1-3, Z2-1, Z2-2, and Z2-3) was extracted (NCBI BioSample No. SAMN10233646). We used the TruSeq RNA Sample Prep Kit (Illumina, San Diego, CA, USA) to get six cDNA libraries for RNA-seq. First, mRNA was enriched by magnetic beads with Oligo (dT); Second, fragmentation buffer was added to cut the mRNA into short fragments; Third, using these mRNA fragments as templates, a single strand of cDNA was synthesized with random primer pd(N)6; Fourthly, buffer, dNTPs, and DNA polymerase I were added to synthesize the ds-cDNA (double-stranded cDNA), and AMPure XP beads were used to purify the ds-cDNA; Fifthly, the purified ds-cDNA was repaired at the end, the A-tail was added, and sequencing adapter was connected; Then, AMPure XP beads were then used to select the 300bp fragment size, and the final six cDNA libraries were obtained after PCR enrichment. The RNA-seq preps were sequenced on the Illumina HiSeq2500 platform (Illumina, San Diego, CA, USA), and the length of sequencing reads was 125bp paired-end (PE125). Clean reads were obtained after the raw RNA-seq sequences (NCBI BioProject No. PRJNA495892) were filtered. After matching the clean reads of six samples with the new gene sequences, the RPKM method was used to calculate the expression of the new genes at the unfertilized egg and prim-5 stage; the new transcripts with significantly decreased expression were screened out at the prim-5 stage.

### PCR validation of new transcripts and expression changes at different developmental stages

mRNA was isolated from Z1-M using an Oligotex mRNA Kit (Qiagen, Hilden, Germany) and full-length cDNA was synthesized by cDNA Library Construction Kit (Takara, Kyoto, Japan). A pair of specific primers (Supplementary Material 1) was designed for each new transcript using the Primer Premier 5 software, and then sent to Sangon Biotech (Shanghai, China) for synthesis. The synthesized full-length cDNA was used as a template for PCR. The sequences could be amplified with the target bands as true transcripts.

Three female and three male zebrafish were used to construct three full-sib groups in a one to one way. 30 individual samples were harvested at unfertilized egg, 2-cell, high, 80%-epiboly, 3-somite, prim-5, and protruding-mouth stages. Samples at the same stage from 3 full-sib groups were mixed together, with seven samples ultimately obtained. For these samples, we used the same methods described above to extract the total RNA and to synthesize cDNA. Using these cDNAs as templates, RT-qPCR was used to detect changes in the expression levels of new transcripts that significantly decreased at the prim-5 stage compared to the unfertilized egg stage. RT-qPCR data were analyzed by two-tailed independent *t*-test. And *P* < 0.05 was considered to be statistically significant.

### Data availability

All Supplementary Materials are as follows. Supplementary Material 1 contain the specific primer pairs of the 84 new transcripts. Supplementary Material 2 contain the sequences of the 84 new transcripts. Supplementary Material 3 contain the annotations of the 84 new transcripts. Supplementary Material 4 contain the prediction and evaluation for the protein-coding potentials of the 84 new transcripts. Supplementary Material 5 contain the expression changes of the 84 new transcripts in the two stages (unfertilized egg and prim-5). Supplementary Material 6 contain the 38 new transcripts that were matched to the datasets obtained by Nudelman *et al.* (Nudelman *et al.* 2018). All the sequencing raw data files were uploaded to the NCBI, and the relevant NCBI numbers are in the corresponding position of this manuscript. Supplemental material available at Figshare: https://doi.org/10.6084/m9.figshare.7837865, https://doi.org/10.6084/m9.figshare.7837931, https://doi.org/10.6084/m9.figshare.7837946, https://doi.org/10.6084/m9.figshare.7837952, https://doi.org/10.6084/m9.figshare.7837964, and https://doi.org/10.6084/m9.figshare.7837973.

## Results

### Insert fragment quality control

A total of 28.14G of clean data, including 1,629,447 polymerase reads, with an average length of 17.13K and an average quality of 0.83, were obtained from the Z1-M PacBio RS II sequencing results after filters were applied. Self-correction of multiple sub-reads generated from the same polymerase reads yielded 954,493 high-quality reads of insert (full passes ≥1, minimum prediction standard ≥0.8). The average insertion sequence length of 0.5-1K libraries was 1,241bp and the quality was 0.9681; the average insertion sequence length of 1-2K libraries was 1,698bp and the quality was 0.9662; the average insertion sequence length of 2-3K libraries was 2,944bp and the quality was 0.9620; the average insertion sequence length of >3K libraries was 4,114bp and the quality was 0.9540.

### Transcripts classification, clustering, and correction

**Figure 1:**
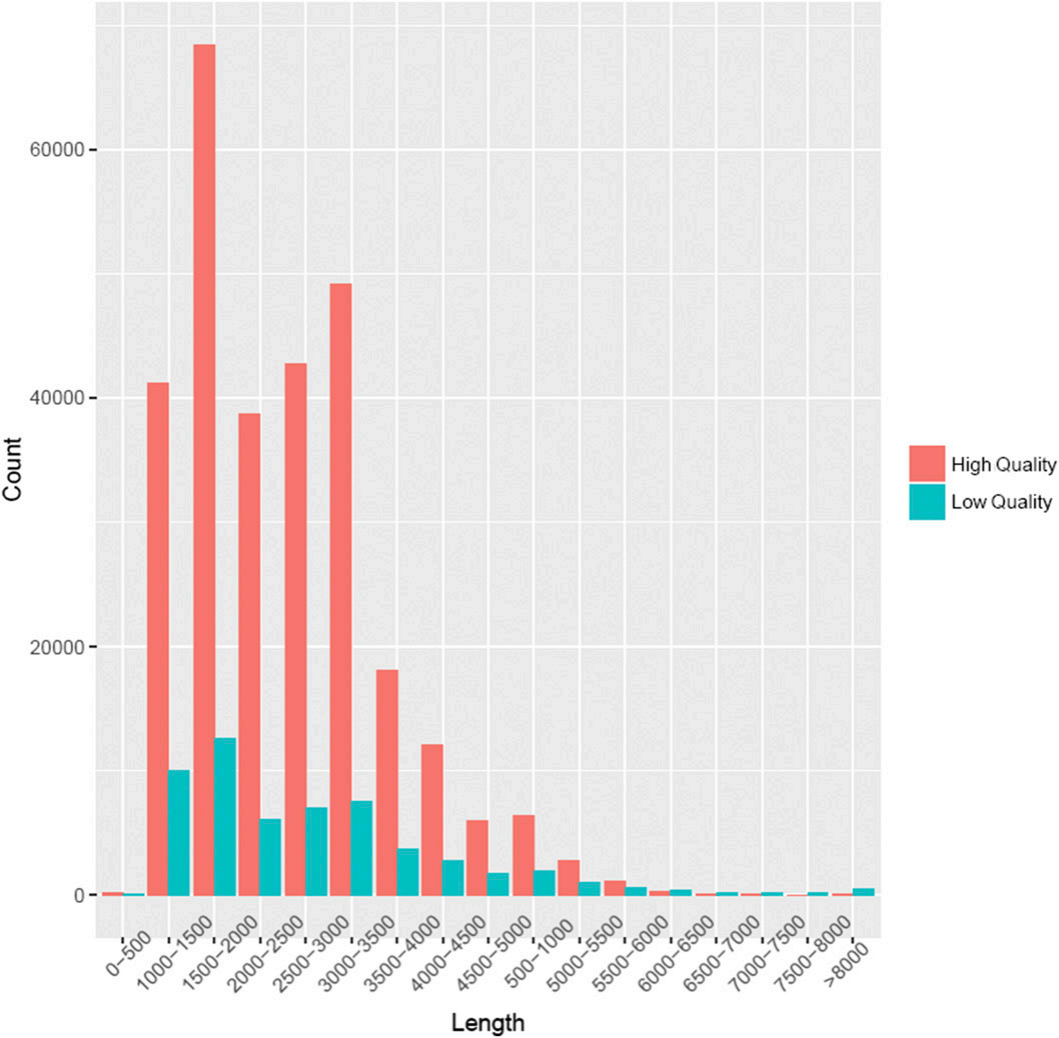
The length distribution of HQ and LQ isoforms. The horizontal axis represents the length, the vertical axis represents the number of isoforms within the length range.

After removing 100,670 (10.55%) <300bp fragments in reads of insert, the transcripts were classified, yielding 274,433 (28.75%) non-full-length reads and 579,390 (60.70%) full-length reads, including 574,407 (60.18%) non-chimerism-full-length reads. The average length of non-chimerism-full-length reads is 1,347bp (0.5-1K), 1,848bp (1-2K), 3,008bp (2-3K), and 4,099bp (>3K). A total of 287,149 HQ isoforms (accuracy >99%) and 56,379 LQ isoforms were obtained by clustering and correcting 574,407 non-chimeric-full-length reads with ICE toolkit. The length distribution map of HQ and LQ isoforms is shown in Figure 1. There is an absolute advantage in both length and quantity for HQ isoforms.

### Matching results of isoforms With reference genome and gene prediction results

A total of 3,970 fusion genes were obtained after HQ and LQ isoforms were matched with the reference genome and 338,541 of the remaining 339,385 isoforms could be matched to the reference genome after removing the fusion genes, accounting for 98.55% of the total number of HQ and LQ isoforms (343,528). After the 338,541 matching isoforms were filtered and redundancy was removed, 102,920 unique isoforms were obtained. 101,019 (98.28%) of the 102,920 unique isoforms could be matched to the zebrafish genome annotation region, mapping 12,782 of the 32,189 annotation genes (including 17,145 transcripts) in the reference genome, accounting for 39.71% of the total annotation genes. The remaining 19,407 genes in the genome were not matched.

**Figure 2:**
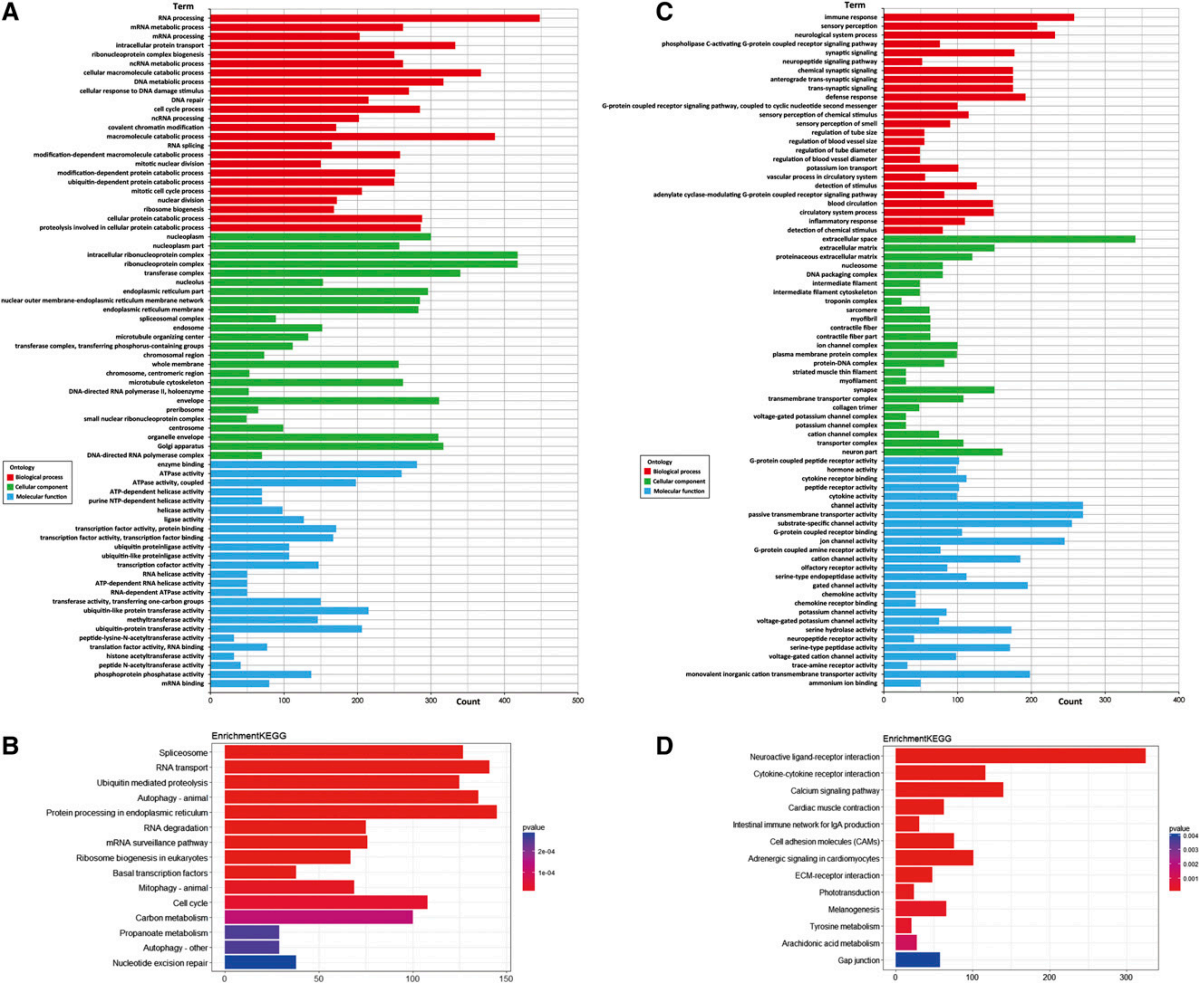
GO and KEGG enrichment of genes. (A) The GO enrichment of 12,782 comparable genes. (B) The KEGG enrichment of 12,782 comparable genes. (C) The GO enrichment of 19,407 incomparable genes. (D) The KEGG enrichment of 19,407 incomparable genes.

As a result of GO analysis of the 12,782 genes that had been sequenced, we found RNA processing, mRNA metabolic process and mRNA processing in biological process; nucleoplasm, nucleoplasm part and intracellular ribonucleoprotein complex in cellular component; enzyme binding, ATPase activity and ATPase activity, coupled in molecular function were highly enriched (Figure 2A). After KEGG analysis, we found that spliceosome, RNA transport, ubiquitin-mediated proteolysis and other pathways were significantly enriched (Figure 2B). GO analysis of the 19,407 genes that had not been sequenced revealed immune response, sensory perception and neurological system process in biological process; extracellular space, extracellular matrix and proteinaceous extracellular matrix in cellular component; G-protein coupled peptide receptor activity, hormone activity and cytokine receptor binding in molecular functionwere highly enriched (Figure 2C). After KEGG analysis, we found neuroactive ligand-receptor interaction, cytokine-cytokine receptor interaction, calcium signaling and other pathways were significantly enriched (Figure 2D). The GO and KEGG enrichment entries of 12,782 genes were not the same as those of the 19,407 genes that had not been sequenced.

Using the ncRNA_pipeline process, 819 ncRNAs were predicted from 1,091 non-annotated isoforms. The remaining isoforms were matched with zebrafish database in NCBI and Ensembl, and 84 new transcripts were screened out (Supplementary Material 2). After annotating using GO, KEGG, COG, SwissProt, TrEMBL, Pfam and NR annotation, 44 new transcripts were preliminarily annotated (Supplementary Material 3). After prediction and evaluation for the protein-coding potentials using CPAT and TransDecoder, 41 new transcripts show coding potentials based on CPAT, but none of them were detected as ORFs with TransDecoder (Supplementary Material 4).

### AS statistics and gene structure optimization

HQ and LQ isoforms were matched with the reference genomes of zebrafish to obtain new gff3 files. After merging with the existing transcripts, only one transcript was detected in 4,823 genes out of 12,782 genes and two or more transcripts were detected out of 7,959 genes. The statistical results of the AS types of 12,782 genes are matched with zebrafish reference genomes in Table 1. The sequencing data identified 40,614 new transcripts of 8,096 genes. A total of 51,675 3′-end or 5′-end elongated regions were obtained after gene structure optimization, supporting 36,347 reads and 9,015 corresponding genes.

**Table 1:** Statistics of alternative splicing (AS) types

TYPE	AS NUMBER	NUMBER OF GENES
ExonS	5,224	2,981
AltD	3,066	2,114
AltA	2,654	1,917
IntronR	9,118	4,367
AltP	8,318	3,779
Other	43,460	7,093

### RNA-seq and preliminary screening of new transcript expression changes

A total of 53,829,566; 61,794,130; 55,833,452; 57,643,214; 70,954,628; and 53,256,284 raw reads were obtained from six samples (Z1-1, Z1-2, Z1-3, Z2-1, Z2-2, and Z2-3) from RNA-seq sequencing. After removing low-quality sequences with conventional filtration, 51,931,870; 59,582,788; 52,514,910; 54,661,228; 65,732,556; and 50,312,762 clean reads were obtained. After matching the clean reads of six samples with the new transcript sequences, the expression levels of the new transcripts at the two stages (unfertilized egg and prim-5) were calculated using the RPKM method. The results of expression are shown in Supplementary Material 5 with 14 new transcripts exhibiting significant decreases in their expression levels at the prim-5 stage as compared to the unfertilized egg stage.

### PCR verification of new transcripts and expression changes in seven developmental stages

PCR validation of all predicted new transcripts that used the cDNA of sample Z1-M as template revealed that 58 of them were found to have transcription products at the unfertilized egg stage (Supplementary Material 5). Among them, 12 new transcripts (PB.689.1, PB.4952.1, PB.5063.1, PB.5289.1, PB.5393.1, PB.5625.5, PB.6621.1, PB.10209.1, PB.10271.1, PB.12107.1, PB.12892.11, and PB.12893.2) exhibiting the greatest decrease in expression levels at the prim-5 stage as compared to the unfertilized egg stage. The annotation results showed that the protein encoded by PB5625.5 contained Glutamate-rich region, the protein encoded by PB12892.11 contained THAP domain, and the protein encoded by PB12893.2 was similar to Nuclease HARBI1 (Supplementary Material 3). It was worth noting that these three transcripts also showed coding potentials based on CPAT (Supplementary Material 4).

**Figure 3:**
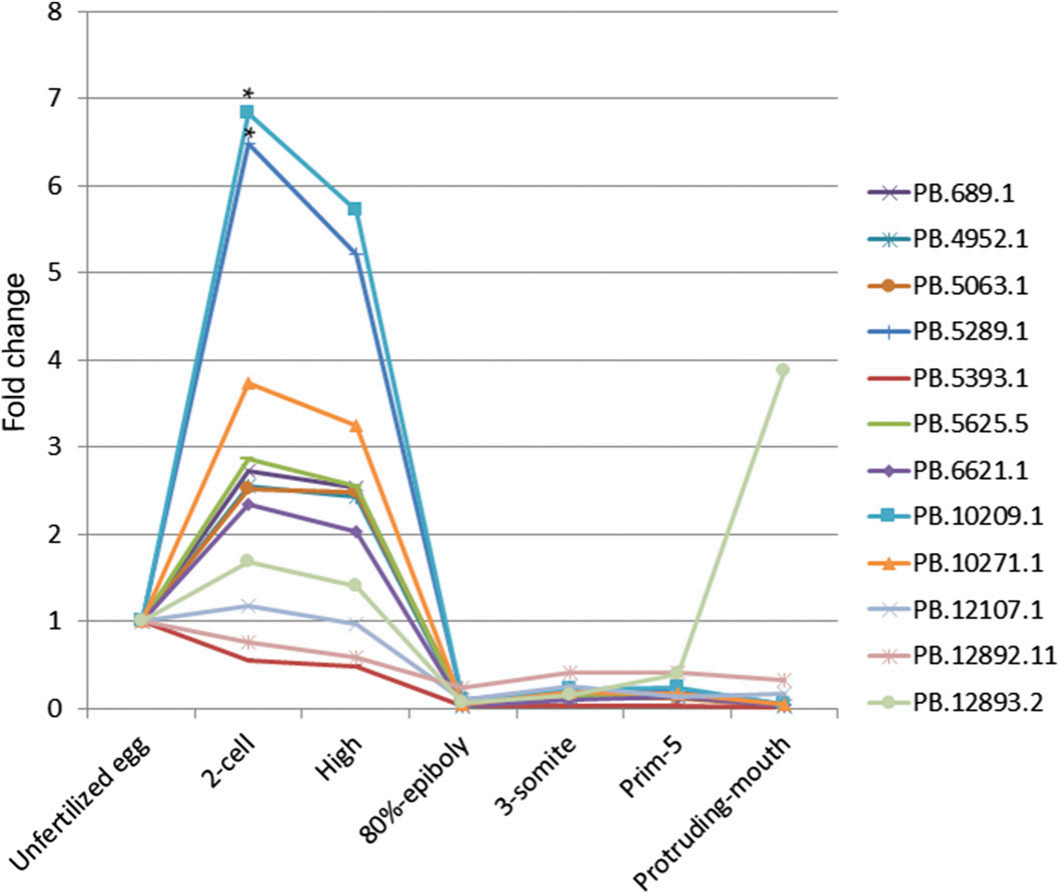
The expression changes of twelve new transcripts at early developmental stages. The horizontal axis represents seven different developmental stages, and the vertical axis represents the relative expression value. “*” indicates that *P* < 0.05.

Subsequently, the expression changes of these 12 new transcripts (PB.689.1, PB.4952.1, PB.5063.1, PB.5289.1, PB.5393.1, PB.5625.5, PB.6621.1, PB.10209.1, PB.10271.1, PB.12107.1, PB.12892.11, and PB.12893.2) at seven developmental stages (including unfertilized egg, 2-cell, high, 80%-epiboly, 3-somate, prim-5, and protruding-mouth) were detected by RT-qPCR (Figure 3). The results showed that these 12 new transcripts exhibited down-regulated expression at the prim-5 stage, as compared to their levels prior to fertilization. Specifically, 10 (PB.689.1, PB.4952.1, PB.5063.1, PB.5289.1, PB.5625.5, PB.6621.1, PB.10209.1, PB.10271.1, PB.12107.1, and PB.12893.2) of these 12 new transcripts were up-regulated expression from the unfertilized egg stage to the 2-cell stage, of which the expression of two transcripts (PB.5289.1 and PB.10209.1) significantly increased by 6.48 and 6.82 times (*P* < 0.05), respectively. While all these 10 new transcripts (PB.689.1, PB.4952.1, PB.5063.1, PB.5289.1, PB.5625.5, PB.6621.1, PB.10209.1, PB.10271.1, PB.12107.1, and PB.12893.2) were down-regulated expression after the 2-cell stage. In these 12 new transcripts, PB12893.2 was up-regulated expression again after the prim-5 stage, while the rest (PB.689.1, PB.4952.1, PB.5063.1, PB.5289.1, PB.5393.1, PB.5625.5, PB.6621.1, PB.10209.1, PB.10271.1, PB.12107.1 and PB.12892.11) remained at low levels; in addition, two transcripts (PB.5393.1 and PB.12892.11) exhibited down-regulated expression during all stages of embryonic development after fertilization.

## Discussion

From the PacBio RS II sequencing performed in this study, we obtained 102,920 unique isoforms, which could be matched with 12,782 of 32,189 annotated genes in the reference genome of zebrafish, accounting for 39.71% of the total annotated genes. The focus of this study was to obtain genes associated with early embryonic development. We only performed full-length transcriptome sequencing using unfertilized eggs. The number of genes captured in this study was relatively less than others have found, which might be mainly related to sequencing samples. For example, Hoang *et al.* represented about 71% of the total number of predicted sugarcane genes from a pooled RNA sample derived from leaf, internode and root tissues (Hoang *et al.* 2017). Wang *et al.* produced 111,151 transcripts from six tissues capturing about 70% of the genes annotated in maize RefGen_v3 genome (Wang *et al.* 2016). This study may not significantly improve the annotation level of zebrafish genome, subsequent sequencing of mixed samples of different tissues may be more useful.

The GO annotations of 12,782 genes matched by the data from PacBio RS II sequencing were enriched in RNA processing, mRNA metabolic processes, mRNA processing, intracellular ribonucleoprotein complex, as well as other functions, primarily genes involved in RNA processing and nuclear protein complexes. KEGG pathways were also enriched in spliceosome, RNA transport, and mRNA surveillance pathways, indicating that RNA splicing, transport, and translation were very active in the early stage of embryonic division. Correspondingly, at the unfertilized egg stage, the 19,407 unmatched genes in the GO enrichment analysis were most significant in immune response, sensory perception, neurological system processes, extracellular matrix, hormone activity, cytokine receptor binding, and the functions mainly related to immune, neurological, and signal transduction. These functions were not enriched in unfertilized eggs, suggesting that these functions are not involved in the division and development of early embryonic cells. The results showed that unfertilized eggs may have abandoned certain secondarily functional maternal genes in order to complete the division in limited space and time, and only retained genes that were involved directly in cell division and development. This is in line with that of Rauwerda *et al.*, who also suggested that the expressed and non-expressed genes showed that maternal mRNA accumulation was a non-random process, as it was related to specific biological pathways and processes relevant in the early embryogenesis stage of unfertilized egg (Rauwerda *et al.* 2016).

As an important mechanism to increase protein diversity and regulate gene expression, AS has attracted the attention of biologists (Vuong *et al.* 2016; Thatcher *et al.* 2016). The detection rate of AS events is related to the detection technology on the one hand and to the species themselves, on the other hand. Due to limitations in sequencing depth and the lack of sensitivity to trace mRNA isoforms, as well as other technological deficits, only about 60% of human genes were estimated to involve AS for a long period of time (Wang *et al.* 2008; Pan *et al.* 2008). In recent years, NGS technology has matured and new NGS sequencing data reveal that about 95% of human genes can be alternatively spliced. (GENCODE Human Annotation, Version 23, http://www.gencodegenes.org). With the rise of third generation sequencing technology (Rhoads and Au 2015), the field of AS has undergone tremendous changes, and our understanding of AS is getting deeper (Cheng *et al.* 2017). We also used third generation sequencing technology to capture 12,782 genes, about 62.27% of which could be alternatively spliced. These data reflect a significantly lower proportion of genes that could involve AS in humans; we speculate that this is related to the evolutionary level of the genomes of the species themselves. The lower level of evolution in fish genomes requiring lesser protein diversity, so the proportion of genes involving AS is also lower.

It was generally believed that there were five main AS types (Zhang *et al.* 2011). In this study, we were able to get statistical results of AS types that could be matched to 12,782 genes in Table 1, with the frequency of IntronR being the highest (19.63%) and AltP, ExonS, AltD and AltA respectively accounting for 16.98%, 13.40%, 9.50% and 8.62%, respectively. According to research results for other species, human ExonS was the main AS type (about 35%), followed by AltA (16%), AltD (15%), and IntronR the least, accounting for only 1% of all AS events (Wang *et al.* 2008). Similar patterns were observed in other mammals, but the frequency of IntronR in plants (such as *Oryza sativa*) is significantly higher, representing the main AS type (Zhang *et al.* 2010). The AS types of zebrafish seemed to be more similar to those in plant; the reasons for this require further study.

Annotation of the zebrafish genome is still being improved. The discovery of new transcripts could not only improve the genome annotation of zebrafish, but also provide new candidates that when functionally investigated may provide new insights into the ZGA of MZT during early embryonic development in zebrafish. In this study, we found 84 new transcripts, of which 58 were indeed transcribed through PCR verification. Recently Nudelman *et al.* sequenced the full-length transcriptome of zebrafish embryos before and after ZGA using PacBio platform (Nudelman *et al.* 2018). Comparing our 84 new transcripts with the datasets obtained by Nudelman *et al.*, we found that 38 of them were matched to the datasets of Nudelman *et al.* (Supplementary Material 6), and the remaining 46 of them were still new transcripts. The embryo samples sequenced in this study were earlier than those taken by Nudelman *et al.* in the embryonic development process. The remaining 46 new transcripts would be very interesting if there were missed because they are only present at the earliest stage of development. Previous researchers have also found NTRs in the annotated and un-annotated regions of the zebrafish genome (Aanes *et al.* 2011; Vesterlund *et al.* 2011; Aanes *et al.* 2013). Aanes *et al.* found an average of 4,067 NTRs in each library, across all chromosomes (Aanes *et al.* 2011). Vesterlund *et al.* identified a total of 3,068 putative NTRs in the 1-cell stage (Vesterlund *et al.* 2011). Aanes *et al.* classified 4,174 of the transcripts as NTRs *i.e.*, possibly new genes (Aanes *et al.* 2013). This analysis indicates that the assembled zebrafish transcriptome contains numerous new transcripts, some of which may be encoded by new genes. NTRs were either found in clusters that are likely to be new genes or represented in close proximity to or within annotated transcripts, probably constituting new exons (Aanes *et al.* 2011). These NTRs have contributed to the improvement of the zebrafish genome annotation. However, there were few new transcripts found in this study, which might be related to the sequencing method. In this study, we used PacBio RS II sequencing, from which sequencing reads were longer and must be completely matched to the un-annotated regions then they could be considered new transcripts. Previous studies used NGS with shorter reads to get sequences; only those reads that matched to the un-annotated regions of the genome were called NTRs, with subsequent tests required for more rigorous validation to prove new transcripts or new genes.

In this study, we found that 12 new transcripts exhibited down-regulated expression at the prim-5 stage, as compared to their levels prior to fertilization. 10 of these 12 new transcripts showed a short-term upward trend from the unfertilized egg stage to the 2-cell stage, of which the expression of two transcripts (PB.5289.1 and PB.10209.1) significantly increased by 6.48 and 6.82 times (*P* < 0.05), respectively; and all these 10 new transcripts were subsequently down-regulated after the 2-cell stage. However, in these 12 new transcripts, the expression of transcript PB12893.2 increased again after the prim-5 stage, while the rest continued to express at low levels. It seems that there is a strong relationship between these 10 new transcripts and the ZGA of zebrafish. The ZGA process generally consists of two stages: one is the starting period of the earliest transcriptional activity, called the activation period of minor ZGA, and the other is the transcriptional period of major ZGA (Lee *et al.* 2014). Major ZGA begins 3hr. after fertilization in zebrafish (Kane and Kimmel 1993; Schier and Talbot 2005). In this study, we found these 10 new transcripts with the highest expression levels at the 2-cell stage (∼45min. after fertilization). Therefore, it was speculated that these 10 new transcripts, especially transcripts PB.5289.1 and PB.10209.1, might take part in minor ZGA, with the function of its expression fulfilled and down-regulated after this period. However, the expression of PB12893.2 increased again after the prim-5 stage, which might still take part in other functions after early embryonic development. The function of these genes in the early embryonic development of zebrafish must be further verified by knockout or inhibition experiments.
